# Differential Expression of lncRNAs in Ovarian Tissue of Meigu Goats During the Sexually Immature and Mature Periods

**DOI:** 10.3390/cimb47060395

**Published:** 2025-05-26

**Authors:** Juntao Li, Yanan Xue, Tao Zhong, Linjie Wang, Li Li, Hongping Zhang, Siyuan Zhan

**Affiliations:** Farm Animal Genetic Resources Exploration and Innovation Key Laboratory of Sichuan Province, Sichuan Agricultural University, Chengdu 611130, China; lijuntao1995@163.com (J.L.); ynanxue@126.com (Y.X.); zhongtao@sicau.edu.cn (T.Z.); wanglinjie@sicau.edu.cn (L.W.); lily@sicau.edu.cn (L.L.); zhp@sicau.edu.cn (H.Z.)

**Keywords:** goat, ovary, RNA sequencing, sexual maturity

## Abstract

The ovary is the primary reproductive organ in goats, and its development significantly influences the sexual maturity and reproductive capacity of individuals. Long non-coding RNAs (lncRNAs) are integral to a wide array of biological processes. However, the regulatory function of lncRNAs in the development of ovarian tissue during sexual maturity in goats remains largely unexplored. In this study, we conducted RNA sequencing on ovarian tissue samples from Meigu goats at sexually immature (3 months, n = 3) and sexually mature periods (6 months, n = 3). We identified a total of 966 lncRNAs across six libraries, with 95 lncRNAs exhibiting differential expression. Additionally, we identified the target genes of these DElncRNAs. Gene Ontology (GO) and Kyoto Encyclopedia of Genes and Genomes (KEGG) enrichment analyses indicated that these target genes were associated with various biological processes and pathways pertinent to ovarian development, including reproduction, reproductive process, JAK-STAT signaling pathway, progesterone-mediated oocyte maturation, Wnt signaling pathway, and cytokine–cytokine receptor interaction. Furthermore, lncRNA–mRNA interaction network analysis suggested that MSTRG.15120.9 and MSTRG.15110.2 play crucial regulatory roles in ovarian development. This study provides a valuable resource for elucidating the molecular regulatory mechanisms of lncRNAs in ovarian tissue during the sexual maturity period in goats.

## 1. Introduction

The Meigu goat, a local breed indigenous to the Liangshan Yi Autonomous Prefecture in Sichuan Province, China, is distinguished by its precocious sexual maturity and high reproductive potential, typically achieving sexual maturity at four to five months of age. The reproductive capacity of goats significantly influences the economic viability of goat production. The ovary is the principal organ responsible for normal reproductive function in goats as it secretes estrogen, which is essential for maintaining sexual characteristics and cyclical reproductive activity [1,2]. Sexual maturity is a pivotal factor in the reproductive performance of goats, with the ovary playing a vital role during this phase by directly regulating the secretion of estrogen and progesterone and sustaining reproductive capability through follicular development and ovulation [3]. The initiation of sexual maturation is a complex process that relies on the integration of various stimuli into neuroendocrine and endocrine signals along the hypothalamic–pituitary–gonadal (HPG) axis. During sexual maturation, there is rapid follicular development, increased ovulation frequency, and enhanced secretion of estrogen and progesterone by granulosa cells [4]. Sexual maturity is widely recognized as being influenced by a variety of complex factors and their interactions, including genetic, metabolic, neuroendocrine, nutritional, and environmental elements [5]. Consequently, sexual maturation represents the primary stage of ovarian development. Nevertheless, the biological mechanisms driving the developmental changes in ovarian tissue during sexual maturation remain inadequately understood.

Long non-coding RNAs (lncRNAs) are RNA transcripts exceeding 200 nucleotides in length, characterized by their complex structure and lack of protein-coding capacity [6]. These molecules play a significant role in regulating gene expression and protein function, thereby contributing to various biological processes. Recent studies have highlighted the involvement of lncRNAs in reproductive processes, such as ovarian development and maturation in female animals [7,8,9,10,11,12]. High-throughput RNA sequencing and functional analyses have been employed to elucidate the reproductive roles of lncRNAs. For instance, one study utilized RNA-seq to investigate the lncRNAs expressed in the ovaries of multiparous and uniparous goats, aiming to explore their functions in ovulation and lambing [13]. Another study demonstrated that the differential regulation of microRNAs (miRNAs) and lncRNAs might be associated with fecundity in Small Tail Han sheep and Dorset sheep [14]. Research conducted by Feng et al. identified five differentially expressed lncRNAs (DElncRNAs) through the analysis of ovaries from Hu sheep with varying reproduction rates, revealing that lncRNAs in sheep ovaries possess regulatory roles in reproduction [15]. Similarly, using comparable methods to study DElncRNAs in black goats, it was suggested that the lncRNAs ENSCHIT00000005909 and ENSCHIT00000005910 positively influence IL1R2, thereby impacting ovarian function [16]. It was reported that lncRNA can be involved in sexual maturation through regulating oogenesis and folliculogenesis [17]. Although lncRNAs play important roles in animal reproduction-related processes, there is very limited information on the functions of lncRNAs during sexual maturation in goats.

In summary, we inferred that lncRNAs played prominent roles in the sexual maturation process of goats, therefore this study conducted an analysis of the lncRNA expression profiles in the ovaries of Meigu goats at three months of age (sexually immature) and six months of age (sexually mature) using RNA sequencing. During these developmental stages, differentially expressed lncRNAs were identified and their functions were subjected to bioinformatics analysis. This study paves the way for further exploring the functions of specifc lncRNAs that may be involved in sexual maturation in goats.

## 2. Materials and Methods

### 2.1. Ethics Statement

The Animal Care and Use Committee of the College of Animal Science and Technology, Sichuan Agricultural University, Chengdu, China, approved all of the animal care, slaughter, and experimental procedures in accordance with the Regulations for the Administration of Affairs Concerning Experimental Animals (Ministry of Science and Technology, Beijing, China) [Approval No. SAU2022302090].

### 2.2. Animal Preparation and Sample Collection

Meigu goats were reared under standard conditions at the Meigu Goat Breeding Farm in Sichuan, China. This experiment was conducted in summer (17~28 °C, relative humidity 72%), and the sexually mature goats were in the anestrus stage. The goats were categorized into two cohorts based on age: 3 months (MG1, n = 3) and 6 months (MG2, n = 3). The MG1 group represents the sexually immature period, while the MG2 group represents the sexually mature period. After slaughter, bilateral ovaries were immediately collected from each ewe and delivered to the laboratory within 2 h. One half of the ovaries was allocated for RNA sequencing and quantitative real-time PCR analysis, and the other half was designated for histological analysis. The ovaries were initially washed twice with 75% ethanol, subsequently immersed in phosphate-buffered saline, and then the ligaments and associated tissues were excised. The isolated ovarian tissue was then frozen in liquid nitrogen and stored at −80 °C. For histological analysis, ovarian tissue samples were fixed in 10% paraformaldehyde. Paraffin-embedded ovary sections were stained using hematoxylin and eosin (H&E) or Masson’s trichrome staining methods. For ELISA detection, blood was collected from the goats by the neck blood collection method and centrifuged at 1500× *g* for 10 min at 4 °C, and the serum was separated. The levels of Gonadotropin-releasing hormone (GnRH), estradiol (E2), progesterone (P4), luteinizing hormone (LH), and follicle stimulating hormone (FSH) were determined using the goat ELISA kit provided by Shanghai Enzyme Linked Biotechnology Co., Ltd. (Shanghai, China).

### 2.3. RNA Extraction, Library Construction, and Sequencing

Total RNA was isolated using Trizol reagent Kit (Invitrogen, Carlsbad, CA, USA) according to the manufacturer’s protocol. RNA quality was assessed on an Agilent 2100 Bioanalyzer (Agilent Technologies, Palo Alto, CA, USA) and checked using RNase free agarose gel electrophoresis. After total RNA was extracted, rRNA were removed. The enriched RNAs were fragmented into short fragments by using fragmentation buffer and reverse transcribed into cDNA with random primers. Next, the cDNA fragments were purified with QiaQuick PCR extraction kit (Qiagen, Venlo, The Netherlands), end repaired, base added and ligated to Illumina sequencing adapters. Then, UNG (Uracil-N-Glycosylase) was used to digest the second-strand cDNA. The digested products were size selected by agarose gel electrophoresis, PCR amplified and sequenced using Illumina HiSeq^TM^ 4000 by Gene Denovo Biotechnology Co. (Guangzhou, China).

### 2.4. Sequencing Data Analysis and lncRNAs Identification

Firstly, the raw data were filtered using the fastp (version 0.18.0) [18] to remove adaptors, reads containing over 10% of poly (N), and low-quality reads. Bowtie2 (version 2.2.8) [19] was used for mapping reads to ribosome RNA (rRNA) database, and the rRNA mapped reads were then removed. The filtered reads were mapped to the reference genome (*Capra hircus*, Ensembl _ release 104) using HISAT2 (version 2.1.0) [20]. The mapped reads from each sample were assembled using StringTie (v1.3.4) [21]. To identify new transcripts, all reconstructed transcripts were aligned against the reference genome using Cuffcompare (v2.2.1) [22]. The transcripts with a classcode “u, i, j, x, c, e, o” were defined as novel transcripts. Next, the transcripts with exon number ≥ 2 and transcript length > 200 bp remained. We then utilized the CPC (v0.9-r2) [23] and CNCI (v2) [24] to predict transcripts with coding potential. Transcripts with a CPC score < −1 and a CNCI score < 0 were eliminated, as well as any transcripts similar to proteins in the Swiss-Prot and Pfam databases (release 33.1). The final lncRNA dataset was derived from transcripts identified as non-coding through the intersection of these two methods.

### 2.5. Differential Expression Analysis

Abundances of the transcripts were quantified using StringTie (v1.3.4) [21]. The expression was normalized by FPKM using RSEM [25]. Differential expression analysis was performed by DESeq2 (version 1.10.1) [26] software between two groups, with |log2 (fold change)| > 1 and *p*-value < 0.05 as the cut-offs for statistical significance. Hierarchical clustering analysis was performed on DElncRNAs using the OmicShare tools (version 3.0), an online platform (www.omicshare.com/tools, accessed on 8 September 2023) for data analysis.

### 2.6. Target Gene Prediction and Enrichment Analysis

Three methods were used to predict target genes for lncRNAs. Cis-target genes of lncRNAs were identified for each lncRNA locus by identifying its 10 kb upstream and downstream protein-coding genes. The trans-regulation of lncRNAs was analyzed by correlation analysis or co-expression analysis of lncRNAs and protein-coding genes, and a Pearson correlation coefficient above 0.95 was considered significant. In addition, RNAplex (v0.2) [27] was used to predict the short interaction between antisense lncRNA and mRNA. Finally, the GO and KEGG enrichment analysis was conducted using the OmicShare tools (version 3.0), an online platform (www.omicshare.com/tools, accessed on 13 September 2023) for data analysis, with the corrected *p* < 0.05 considered significantly enriched.

### 2.7. Construction of lncRNA–mRNA Networks

The target genes of differentially expressed lncRNAs (DElncRNAs) from each comparison were further screened to investigate the interaction between lncRNAs and their target mRNAs. Based on the interaction relationships, potential lncRNAs were filtered and established into visualized lncRNA–mRNA interaction networks using Cytoscape (v3.9.1) [28].

### 2.8. Quantitative Real-Time PCR

Primers for the DElncRNAs (Table 1) were designed using Primer 6.0 software and checked with the NCBI Primer-BLAST tool (https://www.ncbi.nlm.nih.gov/tools/primer-blast/ accessed on 26 September 2023). The expression levels of the lncRNAs were normalized to GAPDH and PGK1. cDNAs were synthesized from DNase-treated RNA using a M5 Super Plus qPCR RT Kit with gDNA Eraser (Mei 5 Biotech, Beijing China). Quantitative real-time PCR analysis was performed with 2× M5 HiPer SYBR Premix Es Taq (Mei 5 Biotech, Beijing China), using a CFX96 Real-Time PCR detection system (Bio-Rad, Hercules, CA, USA). The reaction volume contained 5 μL 2× M5 HiPer SYBR Premix Es Taq, 0.3 μL of 10 μM forward and reverse primers, 1 μL template cDNA, and ddH_2_O to a final volume of 10 μL. The thermal protocol was 95 °C for 30 s and 39 cycles of 95 °C for 5 s; Tm for 30 s. The expression level of lncRNAs were calculated using the 2^−ΔΔCt^ method [29].

### 2.9. Statistical Analysis

The results are expressed as means ± standard error of the mean (SEM). All data were evaluated using Student’s *t*-test to conduct a comparative analysis of two groups by SAS software version 9.2 (SAS, Cary, NC, USA), and differences were regarded as significant at *p* < 0.05 and highly significant at *p* < 0.01.

## 3. Results

### 3.1. Histological Analysis of the Ovaries of Meigu Goats Before and After Sexual Maturity

**Figure 1:**
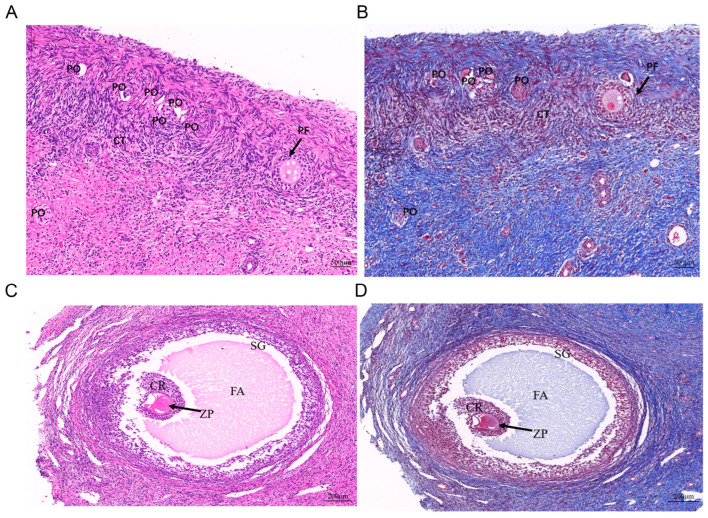
Histological characteristics of Meigu goat ovary. (**A**,**B**) Histological characteristics of the ovary of Meigu goat before sexual maturity (A represents HE staining, B represents Masson staining). Scale bar  =  500 µm. (**C**,**D**) Histological characteristics of mature follicles of Meigu goat (C represents HE staining, D represents Masson staining). Scale bar  =  200 µm. PO: Primary oocyte; PF: Primary follicle; CT: Connective tissue; SG: Stratum granulosum; FA: Follicular antrum; CR: Corona radiata; ZP: Zona pellucida.

In order to understand the ovarian development of Meigu goats during both the sexually immature and mature periods, we conducted a histological analysis of the ovaries. There are a large number of primary oocytes, primordial follicles, and primary follicles distributed in the cortex of the ovarian tissue before sexual maturity (Figure 1A,B). However, after sexual maturity, there are a large number of primary follicles, secondary follicles and a number of mature follicles distributed in the ovary. Mature follicles are composed of theca folliculi, stratum granulosum, follicular antrum, corona radiata, and zona pellucida (Figure 1C,D). Follicles at different stages are important to the reproductive process of livestock. In addition, the ELISA results indicated that the levels of reproductive-related hormones at six months of age were higher than those at three months of age (*p* > 0.05) (Supplementary Table S1).

### 3.2. Overview of lncRNA Sequencing

In total, 14,177,703,050 raw reads were obtained. After filtering out the low-quality and adaptor sequences, 13,973,264,090 clean reads were obtained (Supplementary Table S2). As a result, the percentage of clean reads in all libraries ranged from 98.35 to 98.69%, and approximately 82.07 to 83.41% were uniquely mapped to the *Capra hircus* reference genome across all libraries (Supplementary Table S2). The mean GC content of the six libraries was 50.69%, and the Q30 of each sample was not less than 93.03% (Supplementary Table S2), indicating that the sequencing data were highly reliable and could be used for further analysis.

### 3.3. Identification and Analysis of lncRNAs in Goat Ovary

**Figure 2:**
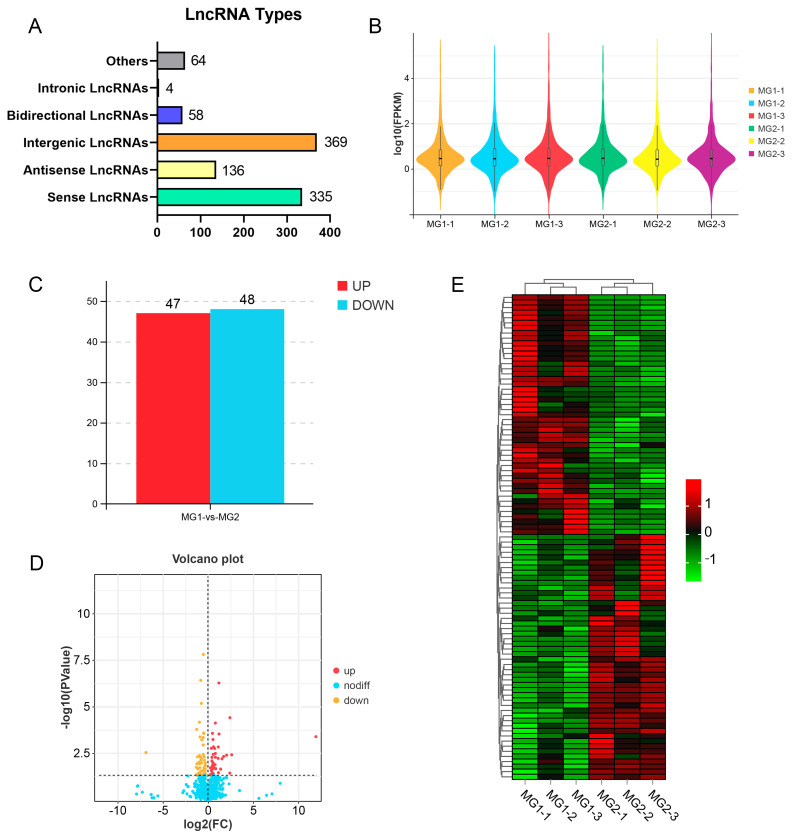
Identification and analysis of lncRNAs in goat ovary. (**A**) Type statistics of identified lncRNAs. (**B**) The six sample expressions in a violin plot, which was replaced by log10(FPKM). (**C**) Numbers of up-regulated and down-regulated lncRNAs. (**D**) Volcano plots showing the up-regulated and down-regulated lncRNAs in MG1 vs. MG2. (**E**) Hierarchical clustering analysis of DElncRNAs. Red: relatively high expression; green: relatively low expression.

RNA sequencing (RNA-seq) identified 966 novel lncRNA transcripts, which comprised 369 intergenic lncRNAs, 335 sense lncRNAs, 136 antisense lncRNAs, 58 bidirectional lncRNAs, 4 intronic lncRNAs, and 64 other lncRNAs (Figure 2A; Supplementary Table S3). To assess the differences among the samples, a violin plot was employed to visualize the expression levels across six samples. The results indicated a high degree of similarity in library construction, sequencing, alignment, and quantification, thereby reinforcing the reliability of the RNA-seq data (Figure 2B) (Supplementary Table S4). Based on our differential analysis, 95 differentially expressed lncRNAs (DElncRNAs) were identified, including 47 up-regulated lncRNAs and 48 down-regulated lncRNAs (Figure 2C,D) (Supplementary Table S5). Furthermore, hierarchical clustering analysis was conducted to evaluate the expression patterns of DElncRNAs and to explore the relationships among the various libraries. This analysis revealed that samples within the same group clustered closely together (Figure 2E).

### 3.4. Enrichment Analysis of Target Genes of DElncRNAs

**Figure 3:**
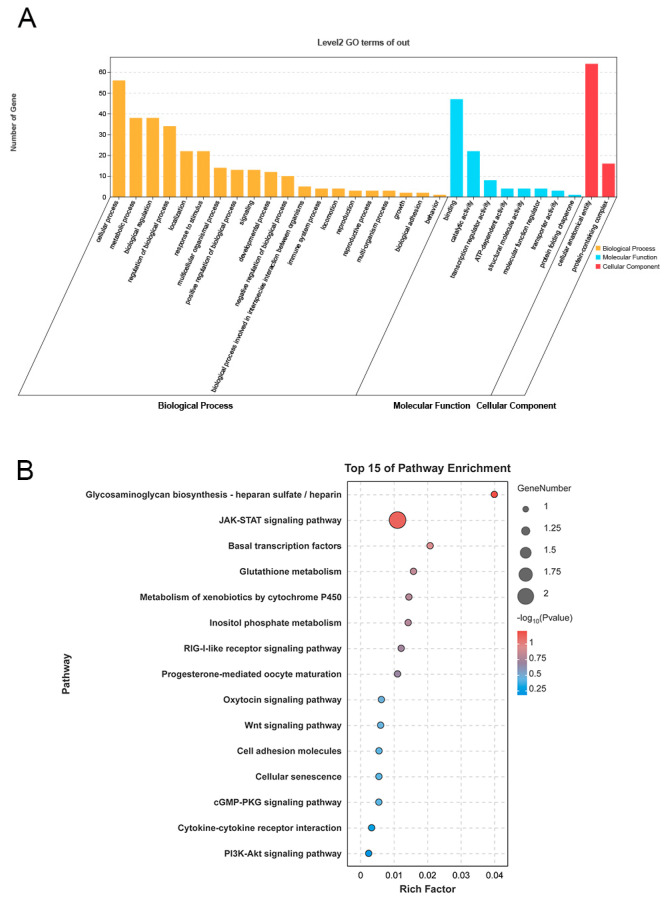
Enrichment analysis of target genes of DElncRNAs. (**A**) The GO analysis of target genes of DElncRNAs. (**B**) The top 15 significant KEGG pathways. The color of the circle represents the Q value. The size of the circle indicates the number of target genes.

In order to explore the potential functions of DElncRNAs, we identified target genes and identified a total of 83 target genes, including 69 cis-target genes, 4 trans-target genes and 10 antisense target genes (Supplementary Table S6). Next, Gene Ontology (GO) analysis of the target genes of DElncRNAs was performed to explore their possible functions. The results showed that the target genes were enriched in 30 GO terms that encompassed a variety of biological processes (Figure 3A) (Supplementary Table S7). Importantly, some of the terms were reproduction-related terms, including reproduction (GO:0000003), reproductive process (GO:0022414), developmental process (GO:0032502), biological regulation (GO:0065007), and regulation of biological process (GO:0050789) (Figure 3A). In addition, the target genes were enriched in 66 KEGG pathways, including JAK-STAT signaling pathway, progesterone-mediated oocyte maturation, Wnt signaling pathway, cGMP-PKG signaling pathway, cytokine–cytokine receptor interaction, and PI3K-Akt signaling pathway (Figure 3B) (Supplementary Table S7). These results indicate that DElncRNAs may be involved in the ovarian development and reproduction process of Meigu goats.

### 3.5. Construction of lncRNA–mRNA Interaction Networks

**Figure 4:**
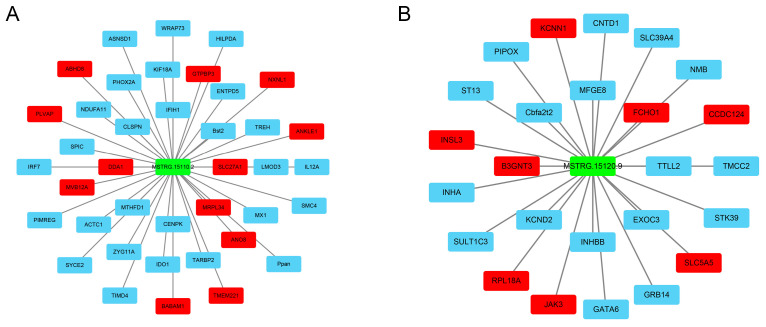
The lncRNA–mRNA interaction networks. (**A**) LncRNA–mRNA networks for MSTRG.15110.2. (**B**) LncRNA–mRNA networks for MSTRG.15120.9. Green represents lncRNAs, red represents the cis-acting interaction, and blue represents the trans-acting interaction. The networks were visualized using Cytoscape (V3.9.1) software, accessed on 20 September 2023.

To explore how lncRNAs interact with target genes to regulate goat ovarian development, we constructed the regulatory networks between lncRNAs and target genes based on the predicted cis and trans-targets using Cytoscape software (v3.9.1). The network analysis focused on two lncRNAs (MSTRG.15110.2 and MSTRG.15120.9) that interacted with more target genes, which probably constitute the center of the network. The number of target genes for MSTRG.15110.2 and MSTRG.15120.9 are 39 and 25, respectively (Figure 4). It is worth noting that these networks include some ovarian development-related genes, such as JAK3, CCDC124, INSL3, INHA, INHBB, etc.

### 3.6. Validation of LncRNAs by qRT-PCR

**Figure 5:**
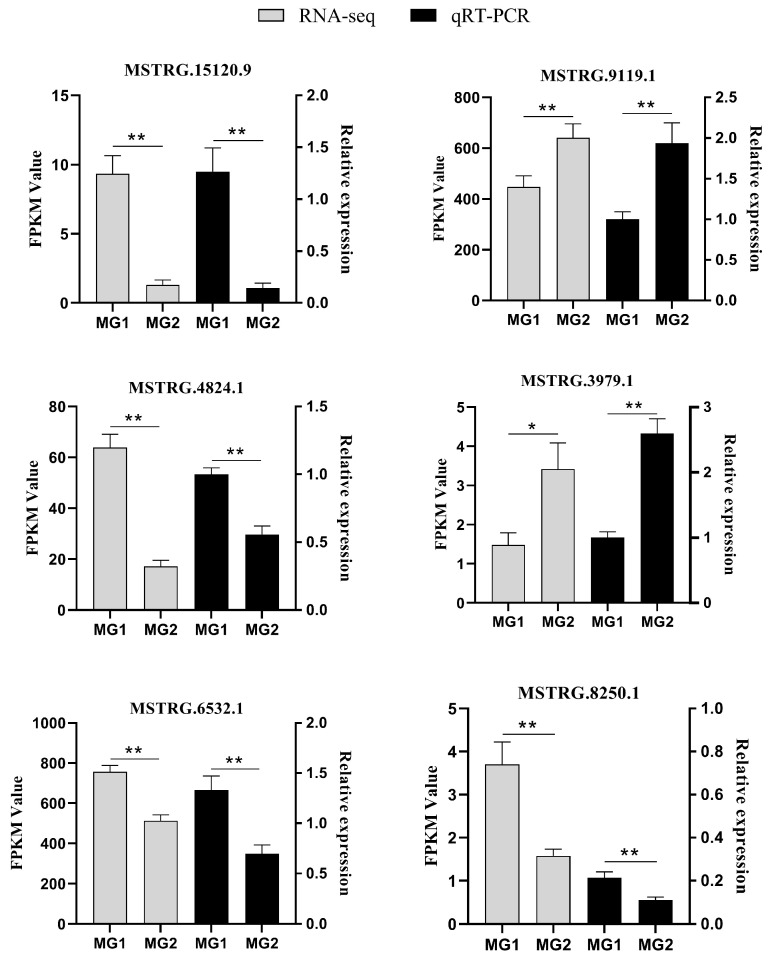
Validation of six DElncRNAs by qRT-PCR. The qRT-PCR data are presented as relative gene expressions, RNA-seq data are presented as fragments per kilobase of transcripts per million mapped reads (FPKM). Data are the mean ± SEM. * *p* < 0.05, ** *p* < 0.01.

To further validate the reliability of the RNA-seq data, six DElncRNAs (MSTRG.15120.9, MSTRG.3979.1, MSTRG.4824.1, MSTRG.8250.1, MSTRG.9119.1, and MSTRG.6532.1) were randomly selected for expression level measurement via quantitative reverse transcription PCR (qRT-PCR). The qRT-PCR results corroborated the expression patterns observed in the RNA-seq data (Figure 5), thereby confirming the accuracy and reliability of the sequencing data.

## 4. Discussion

Sexual maturation represents a pivotal developmental phase in postnatal animals, characterized by substantial morphological and functional transformations in the ovaries, mediated by specific hormones, growth factors, and their receptors [30,31]. Studying the changes in ovarian transcriptome expression profiles during postnatal sexual development in goats is essential for understanding the molecular mechanisms of sexual maturation and reproductive physiology, providing valuable insights for goat breeding. Accordingly, we analyzed the changes in ovarian histological and lncRNA expression profiles at sexually immature (three months) and sexually mature (six months) stages in female Meigu goats. Our research demonstrated notable differences in the phenotypic characteristics of ovaries during the sexually immature and mature periods in Meigu goats. These findings suggest that ovarian morphology undergoes rapid development during sexual maturation, with the continuous formation of mature follicles.

The ovary plays a crucial role in regulating the estrous cycle and fertility in mammals, underscoring its significance as a reproductive organ. An increasing body of research highlights the critical roles of long non-coding RNAs (lncRNAs) in ovarian development across various mammals, including humans [32], sheep [33], pigs [34], cattle [35], and goats [36]. LncRNAs are involved in processes such as ovarian senescence, cell proliferation, and apoptosis, linking them to the regulatory mechanisms of ovarian development in mammals [37,38]. Investigating the role of ovarian lncRNAs during sexual maturation in female goats is crucial for understanding the reproductive mechanisms in this species. In this study, we employed RNA sequencing to identify and analyze lncRNAs in goat ovaries both before and after sexual maturity. A total of 966 lncRNAs were identified, with 95 of these exhibiting differential expression, thereby suggesting their significant roles in goat ovarian development. To investigate the function of lncRNAs in ovarian development both prior to and following sexual maturity in Meigu goats, we identified the target genes of DElncRNAs and conducted functional annotation through GO and KEGG enrichment analyses. Notably, the biological processes “reproduction” (GO: 0000003) and “reproductive process” (GO: 0022414), which are pertinent to reproduction and gonadal development, were enriched. These findings suggest that DElncRNAs enriched in these GO terms may play a regulatory role in the biological processes underpinning ovarian development during sexual maturation in goats.

KEGG enrichment analysis further revealed that the target genes of DElncRNAs were associated with the JAK-STAT, Wnt, and PI3K-Akt signaling pathways, in addition to progesterone-mediated oocyte maturation and cytokine–cytokine receptor interaction, all of which are pivotal in ovarian development, follicle activation, and the regulation of the reproductive cycle. The JAK-STAT signaling pathway plays a crucial role in preserving primordial follicle reserves [39]. Furthermore, JAK signaling is instrumental in the formation of primitive follicles in mice and is involved in the proliferation of granulosa cells; its inhibition, particularly via JAK3, leads to a reduction in granulocyte proliferation. Inhibition of JAK3 expression may also result in impaired formation of primordial follicles [40]. Research indicates that the Wnt signaling pathway is essential for the regulation of normal mammalian reproductive system development. This pathway is primarily involved in the formation of the Müllerian duct, regulation of follicular development, ovulation, luteinization, and the establishment of normal pregnancy [41,42,43,44]. During ovarian development, the Wnt/β-catenin pathway regulates granulosa cell proliferation, differentiation, and apoptotic activity, which are directly linked to follicular development and atresia [45,46,47]. Additionally, the PI3K-Akt signaling pathway is significant in follicular development and facilitates the interaction between oocytes and surrounding cumulus cells [48]. Therefore, the DElncRNAs enriched in reproduction-related pathways plays a crucial role in ovarian development and sexual maturation in Meigu goats.

Furthermore, we developed a network diagram of lncRNAs and mRNAs to elucidate the regulatory influence of lncRNAs on mRNAs. Our analysis identified several target genes, including INSL3, JAK3, and INHA, which are implicated in mammalian reproductive processes. This suggests that the corresponding lncRNAs may have regulatory roles in these processes. For instance, Insulin-like peptide 3 (INSL3), a member of the relaxin family, is associated with the regulation of reproductive functions in mammals [49,50,51]. Notably, INSL3 expression is down-regulated during luteinization induced by the luteinizing hormone (LH) surge prior to ovulation in cattle, indicating its potential involvement in bovine reproduction [52]. Janus kinase 3 (JAK3), part of the membrane-associated intracellular non-receptor tyrosine kinase family, mediates the activation of cytokine and growth factor receptors via the JAK-STAT signaling pathway [53]. Functional studies on bovine endometrial cells have demonstrated that JAK3 enhances the growth of bovine follicles by promoting STAT3 phosphorylation and cellular viability [54]. Additionally, the inhibin alpha (INHA) gene has been linked to litter size in sheep [55] and goats [56,57]. Additionally, the reliability of the lncRNAs identified through RNA sequencing was corroborated by qRT-PCR validation of six randomly selected DElncRNAs. Collectively, these findings suggest that the corresponding lncRNAs perform critical regulatory functions in the ovarian development of Meigu goats.

This study provides important insights into the expression profiles of lncRNAs in the ovaries of Meigu goats before and after sexual maturity, However, it has certain limitations. First, a larger sample size and additional molecular biology experiments are necessary to enhance the reliability and persuasiveness of the results. Future research will focus on unraveling the molecular mechanisms by which lncRNAs regulate sexual maturation in goats at both the molecular and cellular levels. Furthermore, as our analysis was conducted on Meigu goats, it is essential to verify the applicability of these findings to other goat breeds.

## 5. Conclusions

In summary, this study characterized the expression profiles of lncRNAs in the ovaries of Meigu goats before and after sexual maturity. Enrichment analyses revealed that DElncRNAs were linked to a range of biological processes and pathways relevant to ovarian development, such as reproduction, reproductive process, JAK-STAT signaling pathway, progesterone-mediated oocyte maturation, Wnt signaling pathway, and cytokine–cytokine receptor interaction. Moreover, the lncRNA–mRNA interaction networks elucidated in this study provide a valuable resource of candidate lncRNAs involved in ovarian development. This study identified lncRNAs that will help in understanding their regulatory roles in goat ovary development.

## Figures and Tables

**Table 1:** Primer sequences for qRT-PCR validation.

Name	Primer Sequence	Product Size (bp)	Tm (°C)
MSTRG.15120.9	F: GCCTAATGCGACTTCCTAA R: ATACCATCCAGCCATCTCA	188	62.4
MSTRG.3979.1	F: CTCCTCTTCCTGGCTTTC R: TTGGTGGTGGCTTGTTAG	105	56.3
MSTRG.4824.1	F: GCGAATGATTAGAGGTCTTG R: CTTCTCCAACACCACAGT	144	47.7
MSTRG.8250.1	F: CCATCCAACCATCTCATCC R: CCATACTTCAACCACTCAATG	111	47.7
MSTRG.6532.1	F: GGCTAAGATCAAGTGTAGTATC R: CTATTCCAACTCCCTGCTC	106	58.3
MSTRG.9119.1	F: TATACCCTTGACCGAAGAC R: ATCTGGTTGCGACATCTG	181	63.3
GAPDH	F: GCAAGTTCCACGGCACAG R: GGTTCACGCCCATCACAA	249	59
PGK1	F: TGGACCTGTGGGTGTATT R: CTGACTTTATCCTCCGTGTT	159	59

## Data Availability

The results from data analyses performed in this study are included in this article and its tables. The raw sequencing data are available through the NCBI data accession number PRJNA796644.

## Supplementary Materials

The following supporting information can be downloaded at: https://www.mdpi.com/article/10.3390/cimb47060395/s1, Table S1: The content of serum reproductive hormones before and after sexual maturity of Meigu goats; Table S2: Summary of RNA-seq data; Table S3: Summary of lncRNAs identified in the libraries; Table S4: List of lncRNA FPKM results from RNA-seq; Table S5: List of differentially expressed lncRNAs; Table S6: Target genes of differentially expressed lncRNAs; Table S7: Enrichment analysis of target genes of DElncRNAs.
